# Comparative effectiveness and safety of vancomycin versus linezolid for the treatment of central nervous system infections: a meta-analysis

**DOI:** 10.3389/fcimb.2025.1668983

**Published:** 2025-09-18

**Authors:** Liujun Zhou, Qihui Yao, Zecheng Wang, Lingyan Yu, Zhenwei Yu, Yuhua Zhao

**Affiliations:** ^1^ Shaoxing Campus, Sir Run Run Shaw Hospital, Zhejiang University School of Medicine, Shaoxing, China; ^2^ Department of Pharmacy, Second Affiliated Hospital, Zhejiang University School of Medicine, Hangzhou, China; ^3^ Research Center for Clinical Pharmacy, Sir Run Run Shaw Hospital, College of Pharmaceutical Science, Zhejiang University School of Medicine, Hangzhou, China; ^4^ Affiliated Xiaoshan Hospital, Hangzhou Normal University, Hangzhou, China

**Keywords:** vancomycin, linezolid, central nervous system infections, safety, meta analysis

## Abstract

**Objectives:**

This study conducted a meta-analysis comparing vancomycin and linezolid for treating central nervous system (CNS) infections, addressing the lack of comprehensive evaluations in existing research on antibiotic therapy for CNS infections.

**Methods:**

We systematically searched databases, including the PubMed, Embase, Web of Science, Cochrane Library and Chinese databases, up to April 22, 2025. All eligible randomized controlled trials and cohort studies of vancomycin or linezolid were included. The clinical success rate was the primary outcome of interest. The secondary outcomes of interest were cerebrospinal fluid (CSF) parameters, systemic inflammatory markers and the occurrence of adverse drug reactions (ADRs). Two reviewers independently extracted the data and assessed the study quality (NOS/ROB 2.0). The meta-analysis employed random/fixed-effects models to calculate pooled dichotomous outcomes (ORs) and continuous outcomes (SMDs) with 95% CIs via RevMan 5.4.

**Results:**

This meta-analysis included 17 studies (6 head-to-head). Clinical cure rates were not significantly different between vancomycin (84.7%, 222/262) and linezolid (79.7%, 200/251), with a pooled OR of 1.29 (95% CI: 0.55–2.99; p =0.56), while substantial heterogeneity existed (I^2^ = 58%). The secondary outcomes showed no differences but suffered extreme heterogeneity (I² >90%). Safety analysis revealed a significantly greater ADR with vancomycin (21.0% vs. 15.1%; OR 1.63, 95% CI: 1.01–2.65; p = 0.05) with low heterogeneity (I² = 15%).

**Conclusion:**

Vancomycin and linezolid have similar effectiveness in CNS infection from current available evidences, but vancomycin is associated with a greater risk of ADR. Treatment selection should be based on patients’ individual characteristics, such as risk of thrombocytopenia, renal function, and availability of therapeutic drug monitoring.

## Introduction

Owing to high mortality and morbidity rates, central nervous system (CNS) infections impose a substantial clinical burden, representing a significant public health challenge (van de Beek et al., 2016; Tunkel et al., 2017). In CNS infections, gram-positive bacteria predominate, with methicillin-resistant *Staphylococcus aureus* (MRSA) being implicated in a significant majority of cases (van de Beek et al., 2010; van de Beek et al., 2016; Tunkel et al., 2017; Bodilsen et al., 2024). Vancomycin and linezolid are both pivotal for treating CNS infections, but their therapeutic hierarchy remains contested (van de Beek et al., 2016; Tunkel et al., 2017). Vancomycin is endorsed as a first-line therapy by the IDSA/ESCMID guidelines for its bactericidal activities (Tunkel et al., 2017; Bodilsen et al., 2024). However, owing to limited cerebrospinal fluid (CSF) penetration (15–30% of serum levels), aggressive dosing guided by therapeutic drug monitoring (TDM) is necessary to avoid treatment failure (Nau et al., 2010; Liu et al., 2022; Schneider et al., 2022). To achieve adequate CSF or brain concentrations, high doses of vancomycin are recommended. However, these high doses of vancomycin may increase the risk of serious adverse drug reactions (ADRs) (Zamoner et al., 2019; Zamoner et al., 2020). In contrast, linezolid achieves superior CSF bioavailability (70–90%), permitting simplified dosing, but it is restricted to second-line use because of concerns over its bacteriostatic mechanism in CNS compartments and dose-dependent hematologic toxicity (Ntziora and Falagas, 2007; Nau et al., 2010; Tsona et al., 2010; Tunkel et al., 2017; Chen et al., 2020; Pintado et al., 2020; Bodilsen et al., 2024). Moreover, the plasma level of linezolid is lower comparing to vancomycin, thus, its concentration in CSF is also lower than that of vancomycin despite its higher blood-brain barrier permeability.

The lack of comparative evidence makes clinical decision-making for CNS infections challenging. Thus, the aim of this study was to conduct the first systematic review and meta-analysis to evaluate the effectiveness and safety of vancomycin and linezolid for treating CNS infections.

## Methods

### Registration

The study protocol was registered in the International Prospective Register of Systematic Reviews (PROSPERO) with registration number of CRD420251038157. The report is drafted according to PRISMA guideline.

### Literature search

A comprehensive search was performed in PubMed, Embase, Web of Science, the Cochrane Library database, CNKI, WangFang, and Weipu via the keywords “vancomycin”, “linezolid”, “central nervous system infections”, “randomized controlled trial”, and “cohort studies”. The literature review encompassed studies published up until April 22, 2025. The strategy of the PubMed electronic search is presented as an example in **Supplementary Table S1**. In addition, we manually checked the reference lists of the included studies to obtain additional relevant articles. Two investigators independently performed a systematic search of the above database to obtain potentially eligible studies. Any divergence was resolved by the third one.

### Inclusion and exclusion criteria

The inclusion criteria for studies were as follows: (1) patients with confirmed CNS infections; (2) treatment with either vancomycin or linezolid; (3) randomized controlled trials (RCTs) or cohort studies with a control group; and (4) inclusion of at least one effectiveness outcome and/or safety outcome.

The exclusion criteria were as follows: (1) animal or *in vitro* experiments; (2) studies involving patients with tuberculous CNS infections; and (3) Chinese-language articles not indexed in the Peking University Core Journals Directory.

### Data extraction

Two reviewers independently extracted the following information from the included studies: study, nation, study design, study period, treatment, sample size, duration, age, weight, efficacy outcomes, and ADR outcomes. The intervention was structured around a comparative analysis between the control group receiving vancomycin or linezolid and the experimental group treated with alternative antibiotic regimens or combination therapies for CNS infections. Effectiveness and safety-related indicators constituted the outcome measures, incorporating metrics such as the effectiveness rate, changes in clinical indicators such as those in blood and CSF parameters, and the ADR rate.

To ensure methodological rigor, a third reviewer participated in resolving any discrepancies that arose during the data extraction process.

### Outcome of interest

In this meta-analysis, we systematically evaluated the comparative efficacy of vancomycin versus linezolid for managing CNS infections. The clinical success rate, defined as the proportion of patients who were cured or improved at the conclusion of the study, was extracted from the original studies on the basis of their predefined criteria. CSF parameters, including white blood cell (WBC) count, protein quantity, and glucose and neutrophil percentages, were analyzed to assess microbiological and inflammatory responses. Systemic inflammatory markers (peripheral C-reactive protein [CRP] and procalcitonin [PCT]) were also compared between the treatment groups. Additionally, the occurrence of ADRs was assessed to compare the safety profiles of the two antibiotics. This comprehensive approach enables a multidimensional comparison of therapeutic effects, focusing on biochemical resolution, systemic inflammation control, clinical recovery, and general safety in CNS infections.

### Quality assessment

Two independent reviewers conducted methodological quality assessments via standardized tools: the Newcastle–Ottawa Scale (NOS) for cohort studies (evaluating three-domain selection, comparability, and outcome with eight specific items) (Lo et al., 2014) and the Cochrane Risk of Bias 2.0 (ROB 2.0) tool for randomized controlled trials (assessing five critical domains: randomization process, deviations from intended interventions, missing outcome data, outcome measurement, and selective reporting) (Sterne et al., 2019). Each NOS item was scored via a star system (maximum of 9 stars), whereas the ROB 2.0 judgments were categorized as “low risk,” “some concerns,” or “high risk” per domain. Discrepancies in evaluations were resolved through panel discussion or adjudication by a senior methodologist.

### Statistical analysis

The meta-analysis was performed according to the quality of reporting of meta-analyses guidelines and the Cochrane handbook 5.0.1 for systematic reviews of interventions. The Mantel–Haenszel (MH) RR, 95% CI and p value were used to assess efficacy and safety endpoints. Heterogeneity was examined by the chi-square test. Chi-square statistics with a p value < 0.1 were considered to be significant across trials. Treatment effects across trials were combined via a random effects model (I^2^ > 50%) and a fixed effects model (I^2^ < 50%). The meta-analysis was conducted via Review Manager software (version 5.4.1). For categorical data, we computed the summary odds ratio (OR) with a 95% confidence interval (95% CI), whereas for continuous data, the summary standardized mean difference (SMD) with a 95% CI was estimated.

Sensitivity analyses were performed by exclusion of each study one by one to evaluate the stability of results without estimation of bias from individual study.

## Results

### Identification of eligible studies

**Figure 1:**
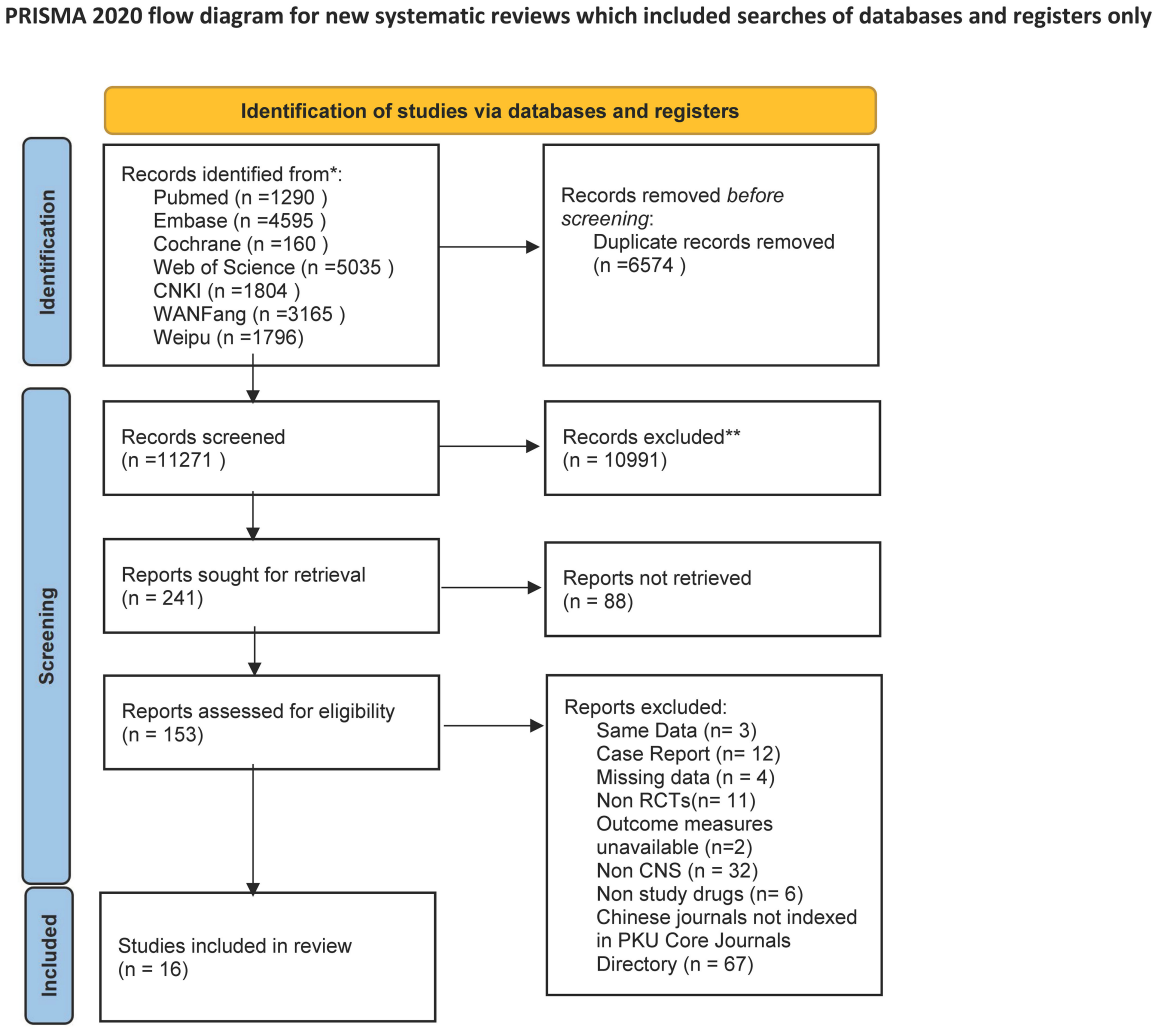
Flow diagram of selection process of the included studies.

The database search retrieved 17,845 records. Due to duplication, 6574 studies were removed. There were 10991 studies marked as ineligible and 88 irrelevant studies. According to the inclusion and exclusion criteria, 153 studies were needed for full-text assessment. Finally, 17 studies were included in the qualitative synthesis. The screening process is shown in **Supplementary Table S1**. Ultimately, 17 studies met our criteria (**Figure 1**) (Qu et al 2018; Huang, 2009; Xu et al., 2010; Wang et al., 2011; Dan et al. 2015; Dai et al., 2016; Xiao et al. 2016; Liu et al., 2016; Wang et al., 2017; Zheng et al., 2017; Cheng, 2018; Yang et al., 2018; Qin et al., 2019; Sun et al., 2024; Yang et al., 2024; Lahouati et al., 2025), including 6 head-to-head studies comparing vancomycin and linezolid.

Owing to the absence of closed evidence loops, noncomparative studies could only be synthesized via single-arm meta-analysis to quantitatively summarize outcome trends (e.g., cure rates, ADR incidence) separately for vancomycin and linezolid.

### Characteristics of eligible studies

The characteristics of the 16 RCTs or cohort studies are described in **Table 1**. The population was mainly Chinese. The included studies used either vancomycin or linezolid. The sample size ranged from 21 to 165, and a total of 1269 participants were included. Among these, the 6 head-to-head studies comparing vancomycin and linezolid included sample sizes ranging from 52-155, totaling 513 participants. According to the mechanism of drug action, we divided the treatment regimens into 6 groups: vancomycin, linezolid, ceftriaxone, norvancomycin, imipenem, no antimicrobial agent and empirical antibiotic therapy (EAT).

**Table 1:** Characteristics of included studies.

No.	Study	Nation	Study design	Study period	Treatment	Disease type	Sample size	Duration, day	Age, year	Weight, kg	Efficacy outcomes^a^	ADR^c^ , n
1	Lahouati (2025)	France (Bordeaux)	Cohort study	2015/01-2023/12	A: Linezolid, IV(1200mg/1800mg, qd) B: Vancomycin, IV	Staphylococcal-Associated CNS Infections	A: 40 B: 51	7d (IQR 4–13)	A:57 (IQR 45-64) B:59 (IQR 48-66)	NR^d^	①	A: 1 B: 9
2	Xiao (2016)	China (Henan)	RCT	2014/01-2015/12	A: Linezolid, IG(600mg, q12h) B: Vancomycin, IG(500mg, qd)	Intracranial Infection after Ventricle-Peritoneal Shunt	A: 35 B: 35	NR	NK	NR	①②③④ ⑥⑦	A: 2 B: 8
3	Qin (2019)	China (Zhejiang)	Cohort study	2015/01-2018/06	A: Vancomycin, IG (1000mg, qd) B: Linezolid, IG (600mg, q12h)	Intracranial Infections after Neurosurgery	A: 33 B: 40	A: 12.7 ± 3.2d B: 9.2 ± 2.5d	A: 53.2 ± 14.9 B: 55.2 ± 13.9	NR	①②③④ ⑥	A: 1 B: 1
4	Sun (2024)	China (Henan)	RCT	2020/09-2022/09	A: Linezolid, IG (600mg, q12h) B: Vancomycin, IG (500mg, qd)	Intracranial Infections after Neurosurgery	A: 36 B: 36	7d	A:40.97 ± 3.43 B:41.16 ± 3.50	A: 22.47 ± 1.29 kg/m² B: 22.52 ± 1.30 kg/m²	①②③④ ⑤⑥⑦	A: 7 B: 6
5	Yang (2018)	China (Shaanxi)	Cohort study	2015/01-2017/11	A: Vancomycin, IG B: Linezolid, IG (600mg, q12h)	Intracranial Infections after Neurosurgery	A: 31 B: 21	A:15.11 ± 6.31d B:14.70 ± 5.12d	A:45.71 ± 10.38 B:47.24 ± 14.22	NR	①②③④ ⑤	A: 2 B: 1
6	Yang (2024)	China (Shandong)	Cohort study	2021/01- 2023/04	A: Vancomycin, IG B: Linezolid, IG	CNS Infections	A: 76 B: 79	NR	A: 43.5 (IQR 14.8-55.0) B: 46.0 (IQR 29.0-57.0)	NR	①	A: 29 B: 26
7	Wang (2017)	China (Hubei)	RCT	2014/01-2017/01	A: Ceftriaxone, IT (0.1g, qd) B: Ceftriaxone, IV (2.0g, q12h) C: Vancomycin, IT (20.0mg, qd) D: Vancomycin, IV (1.0g, qd)	Intracranial Infection during the Perioperative Period of Neurosurgery	A: 20 B: 20 C: 20 D: 20	7d	A: 56.3 ± 11.0 B: 57.1 ± 13.3 C: 55.4 ± 9.8 D: 57.6 ± 12.1	A: 63.3 ± 22.2 B: 62.9 ± 19.0 C: 60.7 ± 24.1 D: 63.1 ± 20.3	①②③④	NR
8	Cheng (2018)	China (Beijing)	RCT	2013/02-2018/02	A: Norvancomycin, IG(0.8g, q12h)+ IT B: Vancomycin, IG(1000mg, qd)+ IT	Postoperative Intracranial Infection after Craniotomy	A: 30 B: 30	A: 28.47 ± 8.74d B: 29.67 ± 9.47d	A: 53.16 ± 11.73 B: 56.23 ± 14.38	NR	①②③④	NR
9	Dan (2015)	China (Baoding)	RCT	2010/03-2014/09	A: Vancomycin, IT(30mg,qd) B: Ceftriaxone, IG(2g, q12h)	Postoperative Intracranial Infection after Craniotomy	A: 43 B: 43	NK	A: 32.2 ± 4.1 B: 32.5 ± 4.5	NR	①②③④	A: 2 B: 3
10	Liu (2016)	China (Zhejiang)	RCT	2011/05 -2014/06	A:Vancomycin, IT B: EAT^b^	Postoperative Intracranial Infection after Craniotomy	A: 40 B: 40	A: 9.45 ± 1.50d B: 22.50 ± 3.50d	A: 45.50 ± 3.50 B: 44.50 ± 2.50	NR	①②③ ④	A: 2 B: 1
11	Dai (2016)	China (Zhejiang)	Cohort study	2015/01-2015/06	A: Ceftriaxone, IT (2 g/d) B: Imipenem, IT (2 g/d) C: Vancomycin, IT (2 g/d)	Postoperative Intracranial Infection after Cerebrospinal Fluid Shunt through Craniotomy	A: 59 B: 46 C: 60	NR	A: 42.15 ± 5.89 B: 39.83 ± 6.01 C: 41.38 ± 6.27	NR	①②	A: 14 B: 1 C: 13
12	Huang (2009)	China (Jilin)	RCT	2008/02-2009/02	A: Vancomycin, IT B: None	Intracranial Infections after Neurosurgery	A: 8 B: 13	A: 5-11d B: 8-17d	NK	NR	①	NR
13	Qu (2018)	China (Hubei)	Cohort study	2015/03-2016/08	A: Meropenem, IV (1mg, q8h) B: Linezolid, IG (600mg, q12h)	Postoperative Intracranial Infections	A: 45 B: 40	A: 2-6d B: 3-8d	A: 39.98 ± 4.12 B: 40.01 ± 4.06	NR	⑥	NR
14	Xu (2010)	China (Shenzheng)	RCT	2001/03-2009/09	A: Vancomycin, IT (50 mg, qd) B: EAT^b^	Postoperative Intracranial Infection after Craniotomy	A: 30 B: 32	NR	A: 40.0 ± 12.5 B: 36.0 ± 14.5	NR	①	NR
15	Wang (2011)	China (Guangxi)	Cohort study	2004/01-2009/01	A: Ceftriaxone, IG B: Vancomycin, IT (50mg,qd)	Intracranial Pyogenic Infection	A: 16 B: 21	A: 20.0 ± 4.7d B: 13.8 ± 3.7d	A: 42 ± 8 B: 48 ± 10	NR	①	A: 0 B: 0
16	Zheng (2017)	China (Zhejiang)	RCT	2013/01-2016/01	A: Vancomycin, IT B: None	Hypertensive Cerebral Hemorrhage Patients with Postoperative Intracranial Infections	A: 40 B: 40	NR	A: 65.1 ± 1.3 B: 65.3 ± 1.5	NR	①⑥⑦	NR

^a^For efficacy outcome:①Clinical success rate; ②cerebrospinal fluid (CSF) blood cell (WBC) count; ③CSF protein quantity; ④CSF glucose; ⑤CSF neutrophil percentage; ⑥C-reactive protein; ⑦procalcitonin.

^b^ETA: empirical antibiotic therapy; ^c^ADR: adverse drug reactions; ^d^NR: Not Reported.

Among these six head-to-head studies, all studies included a baseline assessment, and no significant differences were found in the baseline data.

### Risk of bias

For RCTs, 100% of the studies demonstrated complete outcome data integrity, and 55.6% (5/9) implemented adequate intervention adherence monitoring. However, 44.4% (4/9) exhibited high risk in randomization (Domain 1) due to nontransparent sequence generation, and 44.4% (4/9) had unblinded outcome assessments. Additionally, 22.2% (2/9) showed evidence of selective outcome reporting. The high proportion of studies with critical RoB necessitates cautious interpretation of efficacy estimates.

For cohort studies, the NOS evaluation yielded moderate-to-high quality scores (mean=7.0 ± 0.6): 85.7% (6/7) of the studies lost points in the outcome domain because of insufficient follow-up duration, and a single study (Wang 2011) showed compromised comparability adjustment.

**Figure 2:**
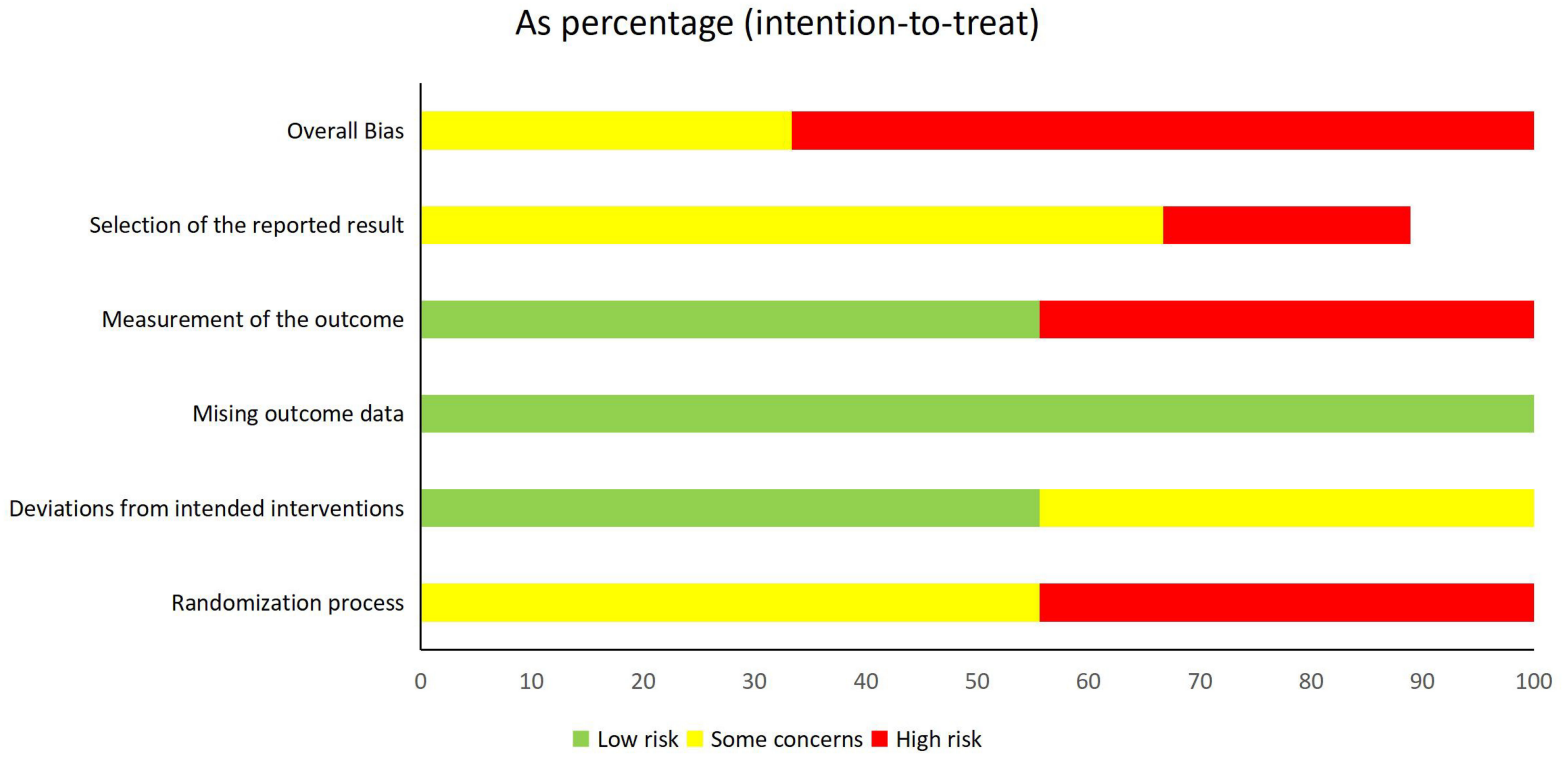
Risk of bias of included RCT.

A visual representation of the risk of bias assessment is provided in **Figure 2** (for RCTs) and **Table 2** (for cohort studies).

**Table 2:** Risk of bias of included cohort studies.

Study	Selection (Max 4)	Comparability (Max 2)	Outcome (Max 3)	Total/9
Lahouati (2025)	4	2	2	8
Qin (2019)	4	2	1	7
Yang (2018)	4	2	1	7
Yang (2024)	4	2	1	7
Dai (2016)	4	2	1	7
Qu (2018)	4	2	1	7
Wang (2011)	4	1	1	6

### Quantitative analysis of effectiveness

#### Clinical success rate

**Figure 3:**
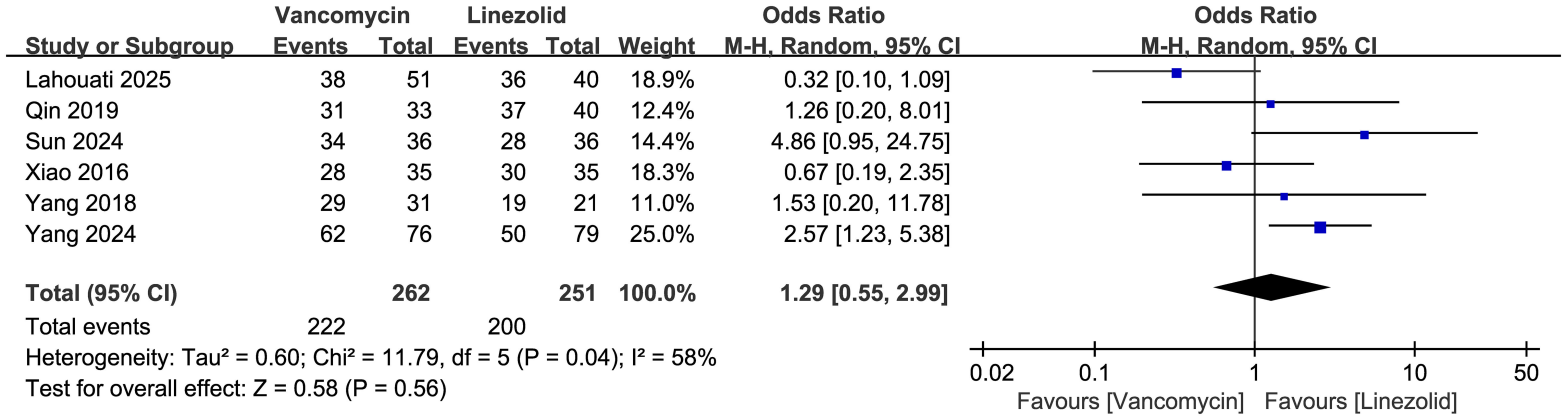
Forest plots of clinical success rate of vancomycin vs linezolid.

Comparisons of the clinical cure rates between vancomycin and linezolid are shown in **Figure 3** (n = 6 studies, 513 patients). A random effects model analysis revealed no significant difference in efficacy, with cure rates of 222/262 (84.7%) in the vancomycin group and 200/251 (79.7%) in the linezolid group. The pooled odds ratio (OR) was 1.29 (95% CI: 0.55–2.99, p=0.56). Substantial heterogeneity was observed (I²=58%, χ²=11.79, df=5, p=0.04).

#### CSF WBC count

**Figure 4:**
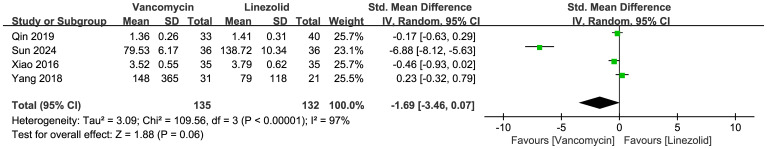
Forest plots of WBC count of vancomycin vs linezolid.

**Figure 4** displays the forest plot comparing CSF WBC counts between vancomycin and linezolid treatments for CNS infections. The random effects meta-analysis of 4 studies (n=267 patients) revealed no statistically significant difference (pooled SMD = -1.02; 95% CI: -2.15 to 0.11; P = 0.06). Extreme heterogeneity was observed (I² = 97%; χ² = 109.56, df = 3, P < 0.00001), primarily driven by the outlier study by Sun et al. (2024), which strongly favored vancomycin (SMD = -6.88, 95% CI: -8.12–5.63).

#### CSF protein quantity

**Figure 5:**
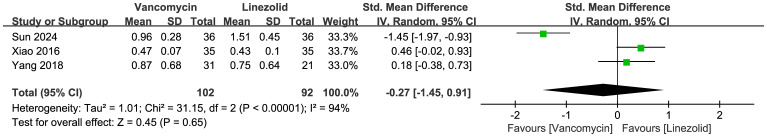
Forest plots of CSF protein quantity of vancomycin vs linezolid.

**Figure 5** presents the forest plot comparing cerebrospinal fluid protein levels between vancomycin and linezolid treatments for CNS infections. The random effects meta-analysis of 3 studies (n=184 patients) revealed no statistically significant difference (pooled SMD = -0.27; 95% CI: -1.45 to 0.91; P = 0.65). Extreme heterogeneity was observed (I² = 94%, τ² = 1.01, χ² = 31.15, df = 2, P < 0.00001), which was primarily attributable to the outlier study by Sun et al. (2024), which strongly favored linezolid (SMD = -1.45, 95% CI: -1.97– -0.93).

#### CSF glucose and CSF neutrophil percentage

**Figure 6:**
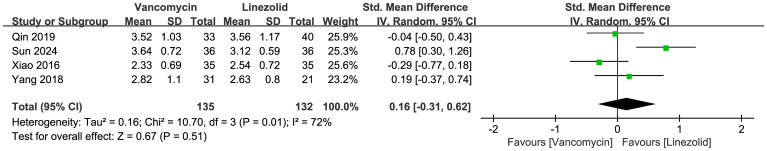
Forest plots of CSF glucose of vancomycin vs linezolid.

**Figure 7:**
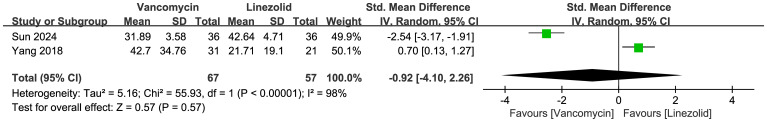
Forest plots of CSF neutrophil percentage of vancomycin vs linezolid.

**Figure 6** presents the forest plot comparing CSF glucose levels between vancomycin and linezolid groups, which showed no significant difference. **Figure 7** presents the forest plot comparing CSF neutrophil percentages between vancomycin and linezolid in CNS infections. The random effects meta-analysis of 2 studies (n=124 patients) revealed no statistically significant difference (pooled SMD = -0.92, 95% CI: -4.10 to 2.26; P = 0.57). Extreme heterogeneity was observed (I² = 98%, τ² = 5.16, χ² = 55.93, df = 1, P < 0.00001), with diametrically opposed effects: Sun et al. (2024) favored vancomycin (SMD = -2.54, 95% CI: -3.17– -1.91), whereas Yang et al. (2018) favored linezolid (SMD = 0.70, 95% CI: 0.13–1.27).

#### CRP

**Figure 8:**
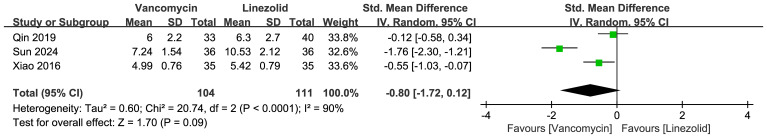
Forest plots of CRP of vancomycin vs linezolid.

**Figure 8** presents the forest plot comparing cerebrospinal fluid C-reactive protein (CRP) levels between vancomycin and linezolid in CNS infections. The random effects meta-analysis of 3 studies (n=215 patients) revealed no statistically significant difference (pooled SMD=-0.80; 95% CI: -1.72–0.12; P = 0.09). Substantial heterogeneity was observed (I² = 90%; τ² = 0.60; χ² = 20.74, df = 2, P < 0.0001), with Sun et al. (2024) demonstrating significantly lower CRP with vancomycin (SMD = -1.76, 95% CI: -2.30 to -1.21)”.

#### PCT

**Figure 9:**
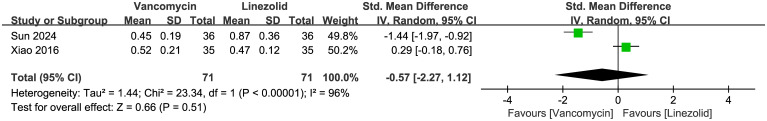
Forest plots of PCT of vancomycin vs linezolid.

**Figure 9** displays the forest plot comparing cerebrospinal fluid PCT levels between vancomycin and linezolid in CNS infections. The random effects meta-analysis of 2 studies (n=142 patients) revealed no statistically significant difference (pooled SMD = -0.57; 95% CI: -2.27–1.12; P = 0.51). Extreme heterogeneity was observed (I² = 96%; τ² = 1.44; χ² = 23.34, df = 1, P < 0.00001), with Sun et al. (2024) reporting significantly lower PCT with vancomycin (SMD = -1.44, 95% CI: -1.97 to -0.92), whereas Xiao et al. (2016) reported a nonsignificant trend favoring linezolid (SMD = 0.29, 95% CI: -0.18 to 0.76).

#### ADR

**Figure 10:**
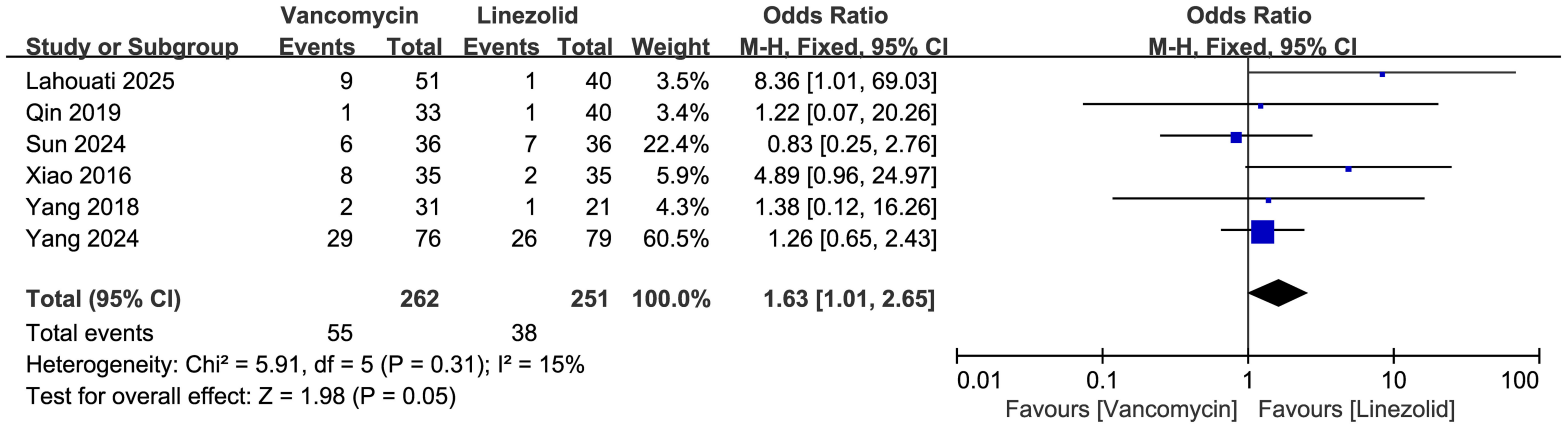
Forest plots of ADR of vancomycin vs linezolid.

Comparisons of the occurrence of ADRs between vancomycin and linezolid are shown in **Figure 10** (n = 6 studies, 513 patients). A fixed-effects model analysis revealed a significant difference in efficacy, with ADR rates of 55/262 (21.0%) in the vancomycin group and 38/251 (15.1%) in the linezolid group. The pooled odds ratio (OR) was 1.63 (95% CI: 1.01–2.65, p=0.05). Low heterogeneity was observed (I²=15%, χ²=5.91, df=5, p=0.31). Among the ADRs reported in these studies, it is noteworthy that vancomycin was associated with four cases of acute kidney injury, and linezolid with two cases of thrombocytopenia.

### Subgroup analysis and sensitivity analysis

Subgroup analysis of clinical success based on study design (RCTs vs. cohort studies) revealed no statistically significant difference in the pooled estimate of clinical efficacy (I² = 0%, χ² = 0.11, df=1, p=0.374). Detailed results are presented in the **Supplementary Material** (**Supplementary Figure S1**).

The sensitivity analyses were carried on by exclusion of individual study one by one. The results of sensitivity analysis of clinical success had shown no substantial difference among the estimates (**Supplementary Figure S2**).

### Qualitative analysis

Among the evaluated antimicrobial regimens, vancomycin demonstrated the most consistent therapeutic outcomes across studies. Pooled analysis of eight single-arm studies (n=267 patients) revealed an overall clinical success rate of 87.2% (95% confidence interval [CI]: 78.4–92.8%).

For ceftriaxone, the aggregated data from four studies (n=143) revealed a pooled success rate of 76.3% (95% CI: 53.1–90.3%).

Norvancomycin, represented by a single study (Cheng et al., 2018, n=30), achieved a 90.0% success rate (95% CI: 73.5–97.9%).

The control groups exhibited divergent outcomes, with empirical antibiotic therapy showing 56.3% success (37.5–75.0%) compared with 89.8% (84.6–95.0%) in the nonantibiotic groups; however, these comparisons should be interpreted with caution due to potential confounding factors.

The safety profiles varied considerably among the interventions. vancomycin-associated adverse drug reactions (ADRs) ranged from 0% to 21.67% across studies, with the highest incidence reported by Dai et al. (2018), primarily involving nephrotoxicity. Ceftriaxone showed a similarly wide ADR range (0–23.73%), with Dai et al. (2016) documenting hepatotoxicity in 23.73% of cases. Notably, imipenem was associated with a 2.17% incidence of seizures in the study by Dai et al. (2017). However, the interpretation of these safety data is limited because six study arms (33.3%) failed to report any ADR outcomes.

The full data are presented in **Supplementary Table S2**.

## Discussion

To the best of our knowledge, this is the first meta-analysis that provides a comprehensive evaluation of the comparative effectiveness and safety of vancomycin versus linezolid for the treatment of CNS infections. The most critical clinical outcome, the clinical cure rate, was not significantly different between vancomycin and linezolid, while a significantly greater rate of ADRs associated with vancomycin than with linezolid.

The pooled analysis revealed cure rates of 84.7% for vancomycin and 79.7% for linezolid, with an OR of 1.29 (95% CI: 0.55–2.99, p=0.56). These findings indicate that both antibiotics achieved similar levels of clinical success in resolving CNS infections within the analyzed studies. Analysis of key CSF markers reflecting inflammation and infection also revealed no statistically significant differences between the two treatment groups. The evaluation of systemic markers also revealed no statistically significant differences.

As for the safety, ADRs occurred in 21.0% (55/262) of vancomycin recipients versus 15.1% (38/251) of linezolid recipients, yielding a pooled OR of 1.63 (95% CI: 1.01–2.65, p=0.05). This finding suggests a potentially less favorable safety profile for vancomycin in this context.

These findings align with but also challenge current guideline recommendations. The IDSA 2017 Guidelines designate vancomycin as first-line therapy for healthcare-associated CNS infections (e.g., postneurosurgical meningitis) owing to its established efficacy against MRSA, while linezolid is listed as an alternative agent when vancomycin is contraindicated. This preference stems from historical concerns about the bacteriostatic (vs. bactericidal) activity of linezolid and its long-term hematologic toxicity (Tunkel et al., 2017). The ESCMID 2024 Consensus similarly prioritizes vancomycin but acknowledges linezolid’s superior CSF penetration (70–90% vs. 30% for vancomycin in inflamed meninges), suggesting that it may be preferable in cases with inadequate cerebrospinal fluid drug concentrations (Bodilsen et al., 2024).

A striking feature across almost all outcome analyses (clinical success rate, CSF WBC, protein, neutrophil %, CRP, PCT) was the presence of extreme or substantial statistical heterogeneity (I² ranging from 58% to 98%). This heterogeneity was frequently driven by the outlier results from a single study (Sun et al., 2024) (Sun et al., 2024). The extreme heterogeneity likely stems from distinct clinical contexts. First, their cohort exclusively comprised postoperative neurosurgical patients with extreme inflammatory states (median baseline CSF WBC >1600×10^6^/L), where enhanced blood–brain barrier penetration may amplify the bactericidal effects of vancomycin (Liu et al., 2022). Second, the assessment at 7 days may have captured the early bactericidal advantage of vancomycin over the static activity of linezolid, particularly in high-burden bacterial infections (Liu et al., 2022). Third, a greater proportion of MRSA infections in the linezolid group (41.7% vs. 38.9%) could skew outcomes if isolates had reduced linezolid susceptibility (Brown et al., 2021). These methodological and population distinctions position Sun et al. as outliers, reflecting acute postoperative management rather than general CNS infection therapeutics.

Therapeutic equivalence supports expanding the role of linezolid, particularly in vancomycin-intolerant patients (e.g., those with renal impairment or refractory thrombocytopenia). The lower adverse drug reaction rate of linezolid (15.1% vs. 21.0%, p=0.05) may favor its use in high-risk populations, despite guidelines not explicitly endorsing this. Dosing optimization: vancomycin’s efficacy relies on aggressive dosing (15–20 mg/kg every 8–12 h) to achieve CSF concentrations >15 μg/mL, a target often missed in real-world settings, whereas linezolid’s consistent CSF exposure (600 mg every12 h) offers practical advantages. Future guidelines should incorporate emerging evidence on comparative safety profiles and consider patient-specific factors (e.g., renal function, concomitant medications) rather than rigidly hierarchizing agents. While Sun suggested the superiority of vancomycin in early postoperative settings, its applicability beyond 7 days and in nonsurgical infections remains unproven. The higher vancomycin concentration (32 mg/L) also raises safety concerns; nephrotoxicity rates may be underestimated in short-term studies (Rybak et al., 2020). Clinicians should weigh these context-specific benefits against linezolid’s consistent CSF penetration and lower renal risk in prolonged therapies.

### Limitations

First, significant methodological heterogeneity existed across studies, with 44.4% (4/9) of the RCTs exhibiting high RoB in randomization and 44.4% (4/9) lacking blinded outcome assessment. This, coupled with inconsistent definitions of ‘clinical cure’ (e.g., microbiological clearance vs. symptomatic improvement), may bias pooled efficacy estimates. Second, all included studies exclusively enrolled Chinese patients, and 73% involved postoperative infections, limiting generalizability to non-Asian populations or community-acquired CNS infections. Third, vancomycin dosing varied substantially (500 mg qd to 1 g q12h) without standardized TDM, whereas unmeasured confounders such as adjunctive carbapenem therapy (29% of cases) or surgical interventions may skew the results. Finally, safety assessments were truncated at ≤14 days, likely underestimating the cumulative hematological risk associated with linezolid.

## Conclusion

This meta-analysis demonstrated comparable clinical efficacy but a less favorable safety profile for vancomycin versus linezolid in treating CNS infections. These findings indicated that treatment selection should be based on patients’ individual characteristics, such as risk of thrombocytopenia, renal function, and availability of therapeutic drug monitoring. Future systematic reviews should also ideally be based on a greater number of studies that are high-quality RCTs comparing standardized regimens (e.g. vancomycin at 15–20 mg/L vs. linezolid at 600 mg every 12 h). There is also a need for research to ensure that antibacterial resistance is not exacerbated by its use.
