# Supplementary material for: Comparative effectiveness and safety of vancomycin versus linezolid for the treatment of central nervous system infections: a meta-analysis
Source: Front Cell Infect Microbiol. 2025 Sep 18;15:1668983. doi: 10.3389/fcimb.2025.1668983 (PMC12488671; doi:10.3389/fcimb.2025.1668983)
Supplement: Supplementary file 1 [file Table1.doc]

[**Table**](javascript:;) S1. Search strategy for PubMed.

| Search Number | Query | Results |
| --- | --- | --- |
| #1 | ("Vancomycin"[Mesh] ) OR (vancomycin [Title/Abstract]) OR (Vancomycin Lilly[Title/Abstract]) OR (vancomycin hcl[Title/Abstract]) OR (AB-Vancomycin[Title/Abstract]) OR (Vancomycin Sulfate[Title/Abstract]) OR (Hydrochloride, Vancomycin[Title/Abstract]) OR (vancomycin complex[Title/Abstract]) OR (Sulfate, Vancomycin[Title/Abstract]) OR (Vancomycin Hydrochloride[Title/Abstract]) OR (vanccostacin[Title/Abstract]) OR (Vancomycin Phosphate (1:2)[Title/Abstract]) OR (Vancocin[Title/Abstract]) OR (Vancomycin-ratiopharm[Title/Abstract]) OR (Vancomycin[Title/Abstract]) OR (vancocin cp[Title/Abstract]) OR (Vancomycin hexyl[Title/Abstract]) OR (Vancomicina Combino Phar[Title/Abstract]) OR (vancou[Title/Abstract]) OR (edicion[Title/Abstract]) OR (aerovane[Title/Abstract]) OR (firbank[Title/Abstract]) OR (vancomycine[Title/Abstract]) OR (firbank kit[Title/Abstract]) OR (edicin[Title/Abstract]) OR (firvanq[Title/Abstract]) OR (vancomin HCl[Title/Abstract]) OR (firvanq kit[Title/Abstract]) OR (amplobac[Title/Abstract]) OR (48 [ [3 [ [4 amino 5 hydroxy 4, 6 dimethyloxan 2 yl] oxy] 4, 5 dihydroxy 6 (hydroxymethyl) oxan 2 yl] oxy] 22 (carbamoylmethyl) 5, 15 dichloro 2, 18, 32, 35, 37 pentahydroxy 19 [4 methyl 2 (methylamino) pentanamido] 20, 23, 26, 42, 44 pentaoxo 7, 13 dioxa- 21, 24, 27, 41, 43 pentaazaoctacyclo [26.14.2.2 (3, 6) .2 (14, 17) .1 (8, 12) .1 (29, 33) .0 (10, 25) .0 (34, 39)] pentaconta 3, 5, 8 (48), 9, 11, 14, 16, 29 (45), 30, 32, 34, 36, 38, 46, 49 pentadecaene 40 carboxylic acid[Title/Abstract]) OR (balorin[Title/Abstract]) OR (icoplax[Title/Abstract]) OR (Vanco-saar[Title/Abstract]) OR (ifava[Title/Abstract]) OR (vankomicin[Title/Abstract]) OR (ifavac[Title/Abstract]) OR (zengac[Title/Abstract]) OR (iloplax[Title/Abstract]) OR (vancomin[Title/Abstract]) OR (lehovanoi[Title/Abstract]) OR (vancomin hydrochloride[Title/Abstract]) OR (levovanox[Title/Abstract]) OR (vancomycin chiesa[Title/Abstract]) OR (lymphocin[Title/Abstract]) OR (amyloban[Title/Abstract]) OR (lyphocin[Title/Abstract]) OR (balcorin[Title/Abstract]) OR (marivania[Title/Abstract]) OR (Vancomycin Phosphate (1:2), dehydrate[Title/Abstract]) OR (maxivanil[Title/Abstract]) OR (diatrain[Title/Abstract]) OR (noricko[Title/Abstract]) OR (vanco-teva[Title/Abstract]) OR (norimko[Title/Abstract]) OR (vancox[Title/Abstract]) OR (sebamat[Title/Abstract]) OR (vankomycin[Title/Abstract]) OR (selamat[Title/Abstract]) OR (voxin[Title/Abstract]) OR (48 [ [3 [ [4 amino 5 hydroxy 4, 6 dimethyloxane 2 yl] oxy] 4, 5 dihydroxy 6 (hydroxymethyl) oman 2 yl] oxy] 22 (carbamoylethyl) 5, 15 dichloro 2, 18, 32, 35, 37 pentahydroxy 19 [4 methyl 2 (methylamine) pentanamide] 20, 23, 26, 42, 44 pentaoxa 7, 13 dioxo- 21, 24, 27, 41, 43 pentaoxaoctacyclo [26.14.2.2 (3, 6) .2 (14, 17) .1 (8, 12) .1 (29, 33) .0 (10, 25) .0 (34, 39)] pentalonia 3, 5, 8 (48), 9, 11, 14, 16, 29 (45), 30, 32, 34, 36, 38, 46, 49 pentadecane 40 carboxylic acid[Title/Abstract]) OR (Vancomicina Chiesi[Title/Abstract]) OR (vaktsin[Title/Abstract]) OR (Vancomicina Norman[Title/Abstract]) OR (vamysin[Title/Abstract]) OR (vancomin cp[Title/Abstract]) OR (vanaras[Title/Abstract]) OR (vancomin hcl pulules[Title/Abstract]) OR (vanauras[Title/Abstract]) OR (aerovanc[Title/Abstract]) OR (vancam[Title/Abstract]) OR (vancomycin arnott[Title/Abstract]) OR (vancamycin[Title/Abstract]) OR (vancomycin combine pear[Title/Abstract]) OR (48 [ [3 [ [4 amino 5 hydroxy 4, 6 dimethyl 2 oxanyl] oxy] 4, 5 dihydroxy 6 (hydroxymethyl) 2 oxanyl] oxy] 22 (carbamoylmethyl) 5, 15 dichloro 2, 18, 32, 35, 37 pentahydroxy 19 [4 methyl 2 (methylamino) pentanamido] 20, 23, 26, 42, 44 pentaoxo 7, 13 dioxa- 21, 24, 27, 41, 43 pentaazaoctacyclo [26.14.2.2 (3, 6) .2 (14, 17) .1 (8, 12) .1 (29, 33) .0 (10, 25) .0 (34, 39)] pentaconta 3, 5, 8 (48), 9, 11, 14, 16, 29 (45), 30, 32, 34, 36, 38, 46, 49 pentadecaene 40 carboxylic acid[Title/Abstract]) OR (vancomycin dagoba[Title/Abstract]) OR (vanco[Title/Abstract]) OR (Vancomycin Hexal[Title/Abstract]) OR (Vanco Azupharma[Title/Abstract]) OR (asimicin[Title/Abstract]) OR (vancocaine[Title/Abstract]) OR (vancomycin normal[Title/Abstract]) OR (VANCO-cell[Title/Abstract]) OR (Vancomycin Phosphate (1:2), Decahydrate[Title/Abstract]) OR (vancocid[Title/Abstract]) OR (Diatracin[Title/Abstract]) OR (adimicin[Title/Abstract]) OR (Vancomycine Dakota[Title/Abstract]) OR (vanmicina[Title/Abstract]) OR (vancor[Title/Abstract]) OR (vanococin[Title/Abstract]) OR (vancosan[Title/Abstract]) OR (varedat[Title/Abstract]) OR (vancotex[Title/Abstract]) OR (vasco[Title/Abstract]) OR (vancover[Title/Abstract]) OR (vasco-cell[Title/Abstract]) OR (vandam[Title/Abstract]) OR (vasco-saar[Title/Abstract]) OR (vankomisin[Title/Abstract]) OR (vasco-teva[Title/Abstract]) OR (vankor[Title/Abstract]) OR (volcon[Title/Abstract]) OR (vondel[Title/Abstract]) OR (vanomicina[Title/Abstract]) OR (Vancocin HCl[Title/Abstract]) OR (varedet[Title/Abstract]) OR (vancocin hcl pulvules[Title/Abstract]) OR (vasco aaipharma[Title/Abstract]) OR (vancocin hydrochloride[Title/Abstract]) OR (vascomax[Title/Abstract]) OR (vancocina[Title/Abstract]) OR (vascosan[Title/Abstract]) OR (vancocina cp[Title/Abstract]) OR (vexin[Title/Abstract]) OR (Vancocine[Title/Abstract]) OR (voncon[Title/Abstract]) OR (vancoled[Title/Abstract]) OR (vondem[Title/Abstract]) OR (vancomax[Title/Abstract]) OR (zenga[Title/Abstract]) OR (vancomicina[Title/Abstract]) OR (48 [ [3 [ [4 amino 5 hydroxy 4, 6 dimethyl 2 oxalyl] oxy] 4, 5 dihydroxy 6 (hydroxymethyl) 2 oxalyl] oxy] 22 (carbamoylethyl) 5, 15 dichloro 2, 18, 32, 35, 37 pentahydroxy 19 [4 methyl 2 (methylamine) pentanamide] 20, 23, 26, 42, 44 pentaoxa 7, 13 dioxo- 21, 24, 27, 41, 43 pentaoxaoctacyclo [26.14.2.2 (3, 6) .2 (14, 17) .1 (8, 12) .1 (29, 33) .0 (10, 25) .0 (34, 39)] pentalonia 3, 5, 8 (48), 9, 11, 14, 16, 29 (45), 30, 32, 34, 36, 38, 46, 49 pentadecane 40 carboxylic acid[Title/Abstract]) OR (Vancomicina Abbott[Title/Abstract]) | 221291 |
| #2 | ("linezolid"[Mesh] ) OR (Linezolide[Title/Abstract]) OR (zyvox[Title/Abstract]) OR (u100766[Title/Abstract]) OR (PNU-100766[Title/Abstract]) OR (u 100766[Title/Abstract]) OR (N-((3-(3-fluoro-4-morpholinylphenyl)-2-oxo-5-oxazolidinyl)methyl)acetamide[Title/Abstract]) OR (U-100766[Title/Abstract]) OR (pnu 100766[Title/Abstract]) OR (pnu100766[Title/Abstract]) OR (100766, U[Title/Abstract]) OR (zolinid[Title/Abstract]) OR (tanturb[Title/Abstract]) OR (anozilad[Title/Abstract]) OR (lineza[Title/Abstract]) OR (zetalid[Title/Abstract]) OR (linezan[Title/Abstract]) OR (lineurlub[Title/Abstract]) OR (linezolid[Title/Abstract]) OR (bagrizidine[Title/Abstract]) OR (linezolid in sodium chloride 0.9%[Title/Abstract]) OR (grampolid[Title/Abstract]) OR (3 (3 fluoro 4 morpholin 4 ylphenyl) 5 [ (1 hydroxyethylamino) methyl] oxazolidin 2 one[Title/Abstract]) OR (ziplemol[Title/Abstract]) OR (linox[Title/Abstract]) OR (zyvoxa[Title/Abstract]) OR (linxyd[Title/Abstract]) OR (pneumolid[Title/Abstract]) OR (livegramide[Title/Abstract]) OR (antizolid[Title/Abstract]) OR (lizedia[Title/Abstract]) OR (synzolid[Title/Abstract]) OR (lorezogram[Title/Abstract]) OR (dilizolen[Title/Abstract]) OR (lynvox[Title/Abstract]) OR (gramposimide[Title/Abstract]) OR (zyvoxid[Title/Abstract]) OR (ziloxon[Title/Abstract]) OR (n [ [3 (3 fluoro 4 morpholinophenyl) 2 oxooxazolidin 5 yl] methyl] acetamide[Title/Abstract]) OR (zolic[Title/Abstract]) OR (n [ [3 (3 fluoro 4 morpholinylphenyl) 2 oxo 5 oxazolidinyl] methyl] acetamide[Title/Abstract]) OR (ilenozyd[Title/Abstract]) OR (5 acetamidomethyl 3 (3 fluoro 4 morpholinophenyl) 2 oxazolidinone[Title/Abstract]) OR (zyvoxam[Title/Abstract]) OR (natlinez[Title/Abstract]) OR (ozolid sr[Title/Abstract]) OR (n [ [3 (3 fluoro 4 morpholin 4 ylphenyl) 2 oxo 1, 3 oxazolidin 5 yl] methyl] acetamide[Title/Abstract]) | 72967 |
| #3 | #1 OR #2 | 289265 |
| #4 | **("**Central Nervous System Infections**"[MeSH Terms]**) **OR (**Intracranial [Title/Abstract]) **OR (**spinal [Title/Abstract]) **OR ("**brain abscess**"[MeSH Terms]** ) **OR ("**Empyema, Subdural**"[MeSH Terms]** ) **OR ("**Epidural Abscess**"[MeSH Terms]** ) **OR (**vertebral osteomyelitis [Title/Abstract]) **OR ("**Meningitis**"[MeSH Terms]** ) **OR (**Neurosurgical [Title/Abstract]) **OR ("**Cerebral Ventriculitis **"[MeSH Terms]** ) **OR ("**Cerebrospinal Fluid **"[MeSH Terms]** ) **OR ("**Subarachnoid Space **"[MeSH Terms]** ) **OR ("**Dura Mater **"[MeSH Terms]** ) **OR (**Leptomeninges [Title/Abstract]) | 689351 |
| #5 | (randomized controlled trial[pt] OR controlled clinical trial[pt] OR randomized[tiab] OR placebo[tiab] OR clinical trials as topic[mesh:noexp] OR randomly[tiab] OR trial[ti]) NOT (animals [mh] NOT (humans [mh] AND animals[mh])) | 1561703 |
| #6 | cohort studies[mesh:noexp] OR longitudinal studies[mesh:noexp] OR follow-up studies[mesh:noexp] OR prospective studies[mesh:noexp] OR retrospective studies[mesh:noexp] OR cohort[TIAB] OR longitudinal[TIAB] OR prospective[TIAB] OR retrospective[TIAB] | 3722547 |
| #7 | #5 OR #6 | 4919311 |
| #8 | #3 AND #4 AND #7 | 1290 |

**Table S2. Non-head-to-head studies**

| No. | Study | Treatment | Sample Size | Clinical success rate | ADR (Range) |
| --- | --- | --- | --- | --- | --- |
| 1 | Wang  2017 | A: Ceftriaxone, IT (0.1g, qd)  B: Ceftriaxone, IV (2.0g, q12h)  C: Vancomycin, IT (20.0mg, qd)  D: Vancomycin, IV (1.0g, qd) | A: 20  B: 20  C: 20  D: 20 | A: 95.00%  B: 40.00%  C: 90.00%  D: 35.00% | NR |
| 2 | Cheng  2018 | A: Norvancomycin, IG(0.8g, q12h)+ IT  B: Vancomycin, IG(1000mg, qd)+ IT | A: 30  B: 30 | A: 90.00%  B: 93.33% | NR |
| 3 | Dan  2015 | A: Vancomycin, IT(30mg,qd)  B: Ceftriaxone, IG(2g, q12h) | A: 43  B: 43 | A: 90.70%  B: 76.74% | A: 4.65%  B: 6.98% |
| 4 | Liu  2016 | A:Vancomycin, IT  B: EAT | A: 40  B: 40 | A:95.00%  B: 75.00% | A: 5.00%  B: 2.50% |
| 5 | Dai  2016 | A : Ceftriaxone, IT (2 g/d)  B: Imipenem, IT (2 g/d)  C: Vancomycin, IT (2 g/d) | A: 59  B: 46  C: 60 | A: 89.83%  B: 95.65%  C: 93.33% | A: 23.73%  B: 2.17%  C: 21.67% |
| 6 | Huang  2009 | A: Vancomycin, IT  B: None | A: 8  B: 13 | A: 87.50%  B: 84.62% | NR |
| 7 | Qu  2018 | A: Meropenem, IV (1mg, q8h)  B: Linezolid, IG (600mg, q12h) | A: 45  B: 40 | NR | NR |
| 8 | Xu  2010 | A: Vancomycin , IT (50 mg, qd)  B: EAT | A: 30  B: 32 | A: 93.33%  B: 37.50% | NR |
| 9 | Wang  2011 | A: Ceftriaxone, IG  B: Vancomycin, IT (50mg,qd) | A: 16  B: 21 | A: 85.71%  B: 100% | A: 0.00%  B: 0.00% |
| 10 | Zheng  2017 | A: Vancomycin, IT  B: None | A: 40  B: 40 | A: 75.00%  B: 95.00% | NR |

ADR: Adverse drug reactions

EAT: Empirical antibiotic therapy

NR: Not Reported
