# Supplementary figures and images for: Comparative effectiveness and safety of vancomycin versus linezolid for the treatment of central nervous system infections: a meta-analysis
Source: Front Cell Infect Microbiol. 2025 Sep 18;15:1668983. doi: 10.3389/fcimb.2025.1668983 (PMC12488671; doi:10.3389/fcimb.2025.1668983)

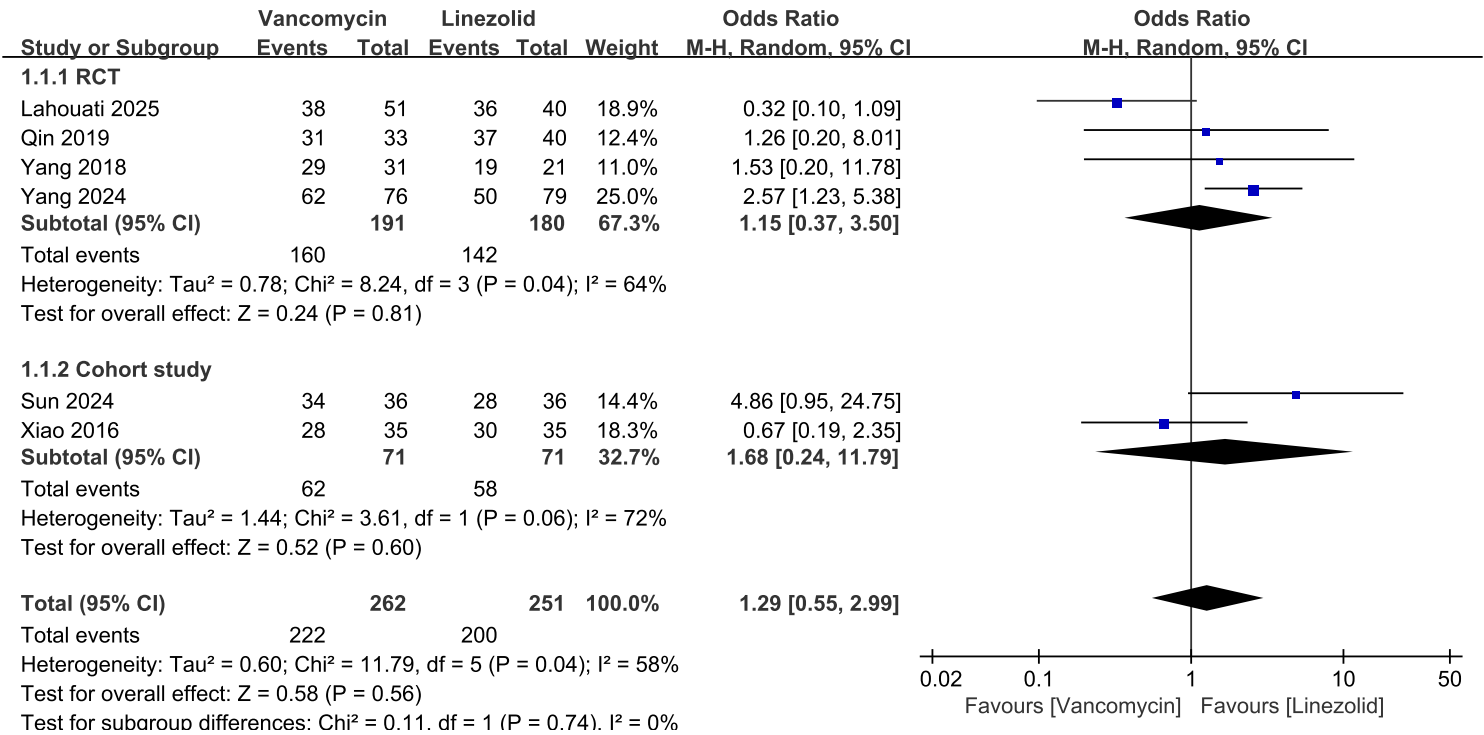

Supplement: Supplementary file 2 [file Image1.pdf]

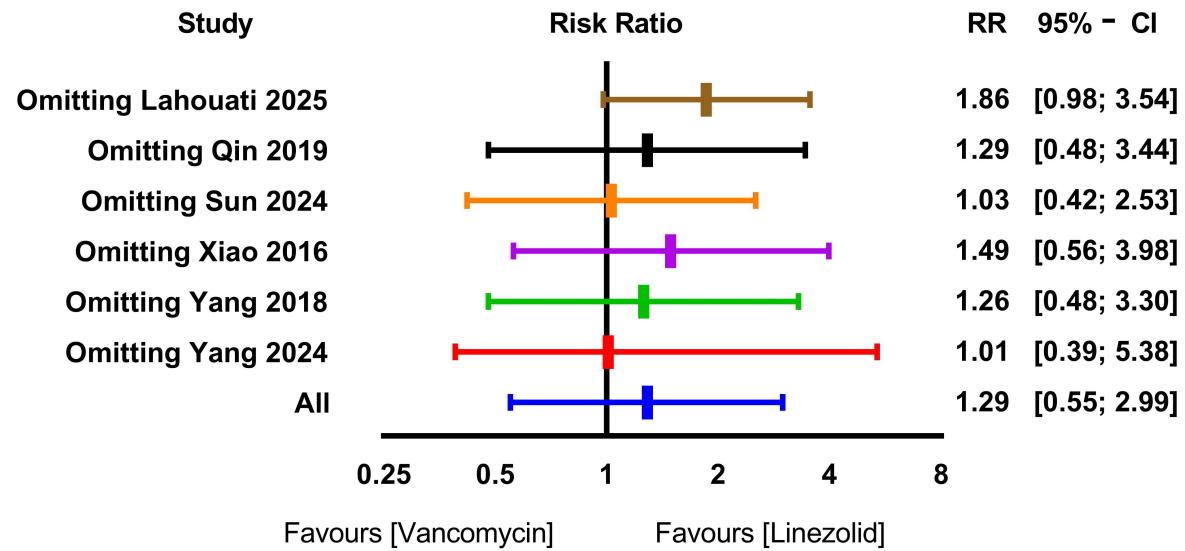

Supplement: Supplementary file 3 [file Image2.pdf]
